# Use of Heated Tobacco Products and Pulmonary Function in the Real World: More Studies Needed to Answer Many Important Questions

**DOI:** 10.2188/jea.JE20210418

**Published:** 2022-04-05

**Authors:** Harumitsu Suzuki, Akira Fujiyoshi

**Affiliations:** Department of Hygiene, Wakayama Medical University, Wakayama, Japan

Heated tobacco products (HTPs) emit less (potentially) harmful constituents than combustible cigarette because, unlike conventional cigarette smoking, HTPs heat tobacco without burning it. Based on this reduced emission, tobacco industries advertise HTPs as more desirable for health than conventional cigarettes, although evidence is yet to be established with regard to impact of HTP use on clinically meaningful health outcomes. Most of the current literature on HTPs described only short-term relationships with surrogate markers of health outcomes.

A HTP, produced by Philip Morris, was introduced into the Japanese market in 2014, ahead of other countries, and its sale has been on the rise ever since. Now, many brands of HTPs are available globally. Given the uncertain health impact of HTPs, it is an important first step to know how common HTP users and “dual users” (ie, those who use HTPs and smoke cigarettes) are in a community. However, the relevant literature is limited, and the estimated prevalence of HTP users and that of dual users remained unclear with conflicting reports. For example, the Japan “Society and New Tobacco” Internet Survey (JASTIS) and International Tobacco Control (ITC Japan),^1^^,^^2^ both using internet surveys, showed that the majority of HTP users were concurrent cigarette smokers. On the other hand, the Japanese National Health and Nutrition Survey in 2019 reported the opposite trend.^3^ A potential source of such discrepancy may be the sole reliance on self-report in assessing HTP use in those surveys. In contrast, Kinjo et al estimated HTP use using interviews, but they did not separate dual users from overall HTP users in their report (ie, their age-adjusted prevalence of “HTP smoker or dual user” were 8.3% in men and 1.8% in women, based on 4,628 participants in a nationwide survey in Japan).^4^

In this issue of the Journal,^5^ Harada et al report prevalence of HTP-only users and dual users based on the assessment of face-to-face interviews by trained interviewers, from a residential population and a worksite population in Japan. According to the report, crude sex-combined prevalence was 0.8% for HTP-only users and 0.6% for dual users in the residential population (*n* = 2,612; mean age, 67.7 years; 54.9% women) in 2018–2019. The corresponding prevalence for HTP-only users and dual users was 5.0% and 1.9%, respectively, in the worksite population (*n* = 722; mean age, 49.3 years; 65.4% women). Having estimated the total number of tobacco products smoked/used per day at baseline (2012–2015) and at follow-up (2018–2019), the authors observed that, among other smokers (ie, cigarette-only and HTP-only), only dual users increased their consumption of tobacco products over the course of follow-up (although estimated amount of total tobacco products consumed were not provided for each smoking group). This finding casts serious doubt on the notion that HTPs are useful for “harm reduction” as advertised by tobacco industries^6^ because the finding indicates that some smokers tend to consume greater amount of tobacco products, HTPs and cigarettes combined, when HTPs become available to them in a real-world setting.

Another novel and potentially concerning finding from Harada et al’s paper is that annualized decline in forced expiratory volume in one second (FEV_1_) was the greatest by point estimate in dual users (−63 mL/year, *n* = 16), among current and past smokers (−34 to −44 mL/year, *n* = 1,061), and in never smokers (−31 mL/year, *n* = 1,535). The decline in FEV_1_ of the dual users was significantly greater than that of cigarette-only smokers (−44 mL/year, *n* = 233).^5^ To our knowledge, this is the first study showing longitudinal change in FEV_1_ among HTP users in a real-world setting, presented by a group of researchers unrelated to the tobacco industry. Although FEV_1_ itself is a surrogate of lung disease, its rapid decline suggests progression of airway obstruction, the known pathological change related to cigarette smoking. The greater decline in FEV_1_ observed in Harada et al’s paper seems inconsistent with three previous studies, all of which were affiliated with tobacco companies, reporting that combustible cigarette smokers who switched to HTP use were shown to have better %FEV_1_ (ie, a proportion of measured FEV_1_ divided by one predicted according to age, sex, and height) as compared to those who continued to smoke combustible cigarette.^6^^–^^8^ The reason for the inconsistency between Harada et al’s result versus those industry-related reports are not entirely clear, but may be due to difference in follow-up period (≈1.5 years after starting HTPs in Harada et al’s study vs up to months in the industry-related papers), and difference in methodology of pulmonary function test, including use or non-use of bronchodilators (some of the industry-related papers used bronchodilator, whereas Harada et al’s paper did not). The greater decline in FEV_1_ observed in the dual users in Harada et al’s study may be explained in part by their greater consumption of total tobacco products, as discussed above. Consistent with this explanation, statistical adjustment for number of tobacco product at follow-up attenuated the observed difference toward the null. If this finding can be replicated in other settings, HTPs may not be helpful for “harm reduction” in a real-world setting because, again, a subgroup of HTP users may consume more tobacco products leading to more rapid progression of airway obstruction in the lungs.

HTP users tended to be younger than cigarette-only users, which is consistent with previous reports. According to a separate study, a higher proportion of HTP users believed that “HTPs are much or somewhat less harmful than cigarettes” and that “secondhand emissions from HTPs are much or somewhat less harmful than those from cigarettes” compared to exclusive smoker groups.^9^ Such beliefs may be a basis for preferring HTPs to conventional tobacco in some people. The same study also reported dual users being younger and wealthier than exclusive smokers.^9^ In contrast, the current study did not provide a clear characterization of dual users: they were statistically not different from other smokers with regard to level of nicotine dependency and stage of readiness for smoking cessation.

Limitations of Harada et al’s study include a small sample size of HTP users, especially those who provided FEV_1_ data. Another limitation was lack of provision of sex-specific results in the main analyses. It would have been more informative if the results were presented by sex strata because, in general, smoking behavior is very different by sex. Likewise, it would have been more informative if other standardized measures of lung function, such as %FEV_1_ or ratio of FEV_1_ to Forced Vital Capacity (FVC) (measured FEV_1_ divided by measured FVC), were presented along with FEV_1_ because both FEV and FEV_1_ are influenced by sex, height, and age. Lastly, in their study population, no participant was a *de novo* HTP users, and all the HTP users had a history of smoking. Therefore, it seems difficult to judge to what extent the observed decline in FEV_1_ in HTP users should be attributed to HTP use rather than previous cigarette smoking.

In conclusion, Harada et al’s study added important pieces of information to the literature of HTPs: prevalence of HTP users, including dual users, in residential and worksite populations in Japan using face-to-face interview by trained interviewers. The observed annualized decline in FEV_1_ of dual users, greater than cigarette-only users, was concerning but needs replication with a larger sample size along with total amount exposure to tobacco products. Since industry-related studies are common in the HTP literature,^10^ we call for more independent research in this emerging field to answer many important questions about HTPs. As a next step, we suggest investigating prevalence and lung functions of *de novo* HTP users relative to other groups of smokers and non-smokers according to sex and age strata. Also, characterization of dual users (“who is likely to use HTPs and smoke cigarettes?”) remains important. Ultimately, risk of clinically meaningful health outcomes must be examined among HTP users, including dual users, from a longitudinal observational study.
